# Investigating the trends of incidence rates of breast cancer in Southern Iran: a population based survey

**DOI:** 10.1186/s12905-023-02757-7

**Published:** 2023-11-10

**Authors:** Sezaneh Haghpanah, Mahnaz Hosseini-Bensenjan, Mani Ramzi, Zahra Khosravizadegan, Abbas Rezaianzadeh

**Affiliations:** 1 Hematology Research Center, Shiraz University of Medical Sciences, Shiraz, Iran; 2 Fars Population‑Based Cancer Registry, Shiraz University of Medical Sciences, Shiraz, Iran; 3 Colorectal Research Center, Shiraz University of Medical Sciences, Shiraz, Iran

**Keywords:** Age group, Breast cancer, Incidence trend, Morphology, Topography

## Abstract

**Background:**

The overall incidence of breast cancer is different all over the world and even within a nation. The present study aims to investigate the stratum-specific incidence trends of breast cancer in southern Iran.

**Methods:**

In this retrospective cohort study, the data of Fars Population-Based Cancer Registry was used during 2001–2018. New cancer cases with ICD-O-3 codes C50.0 to C50.9 were categorized based on age group, morphology, and topography. Age-specific incidence rates of breast cancer were calculated during 2001–2018. Annual overall and truncated age-standardized incidence rates and their 95% Confidence Intervals (CIs) were also calculated. Afterward, the Annual Percentage Changes (APCs) of the age-specific and age-standardized incidence rates of breast cancer during 2001–2018 were calculated using Joinpoint regression software.

**Results:**

An increasing trend was observed in the incidence of breast cancer among women during 2001–2018 (APC of age-standardized incidence rates: 9.5 (95% CI: 7.5, 11.5)).However, the trend was increasing less during the recent years. The APC of age-standardized rates decreased from 15.03 (95% CI: 10.4, 19.8) in 2007 to 6.15(95% CI: 4.0, 8.4) in 2018. The most common morphology of breast cancer was invasive ductal carcinoma (77.3% in females and 75.1% in males) and its trend was similar to the general trend of different types of breast cancer. The most common site of breast cancer was the upper outer quadrant. Most breast cancer cases were female and males accounted for 2.45% of the cases. Among females, 40–55 was the most prevalent age group.

**Conclusion:**

The incidence of breast cancer among women living in southern Iran showed an increasing trend from 2001 to 2018. However, the rate of increase exhibited a milder slope during the more recent years. Based on the higher prevalence of breast cancer in the 40–55 age group observed in the present study, it offers valuable insight into the potential reduction of the breast cancer screening age from 50 to 40 years for healthy Iranian women. However, before implementing such a policy change, it is crucial to conduct additional studies that specifically examine the cost-effectiveness, as well as the potential benefits and risks associated with this alteration.

**Supplementary Information:**

The online version contains supplementary material available at 10.1186/s12905-023-02757-7.

## Introduction

Breast cancer is the second most commonly diagnosed cancer in women following lung cancer [1]. According to histological appearance, breast carcinoma is classified into ductal or lobular adenocarcinoma [2]. Most breast malignancies are adenocarcinoma, accounting for more than 95% of cases [3].Invasive breast cancers refer to a heterogeneous group of tumors, which are mainly classified as ductal and tubular breast carcinoma. Among invasive ones, Invasive Ductal Carcinoma (IDC) is the most common type [4].

The incidence of breast cancer is higher in developed countries, and it is the most fatal cancer in the female population [1]. There are modifiable risk factors for breast cancer including obesity [5], physical inactivity [6], western diet [7], alcohol consumption [8], smoking [8], and using oral contraceptive pills [9]. During the past decades, the prevalence and distributions of the main risk factors for cancer have changed globally [1, 10].

Similar to other cancers [11, 12], the overall incidence of breast cancer is different over the world and even within a nation. These variations are more significant regarding age group, topographical location of the tumor, and morphological subtype [13].

Investigating the trends in the incidence and mortality of breast cancer in the United States (US) from 2009 to 2018, along with a review of the recently reported census worldwide, reveals an increasing trend in the incidence rate without a corresponding increase in the mortality rate. This can be attributed to improved screening practices and better clinical management of patients [14]. However, it is crucial to prioritize breast cancer in developing and poorer countries due to limited resources, which often result in challenges with screening, late-stage diagnosis, and less efficient treatment [14, 15]. Additionally, in these regions, distinct epidemiological features have been observed compared to Western countries. These include a younger age at onset, a more aggressive form of breast cancer, and a higher frequency of certain rare forms such as inflammatory breast cancer or male breast cancer. These unique characteristics highlight the need for increased focus and attention on addressing these specific challenges in breast cancer management within these regions [15]. The present study aims to investigate the stratum-specific incidence trends of breast cancer in southern Iran considering age and morphological subtypes.

## Method

In this retrospective cohort study (Supplementary Table 1), the data of Fars Population-Based Cancer Registry (FPBCR) was used for breast cancer incidence during 2001–2018. This registry was founded in 2001 as a pathology-based registry and was then promoted to a population-based system in 2007. Shiraz, the capital of Fars province located in southern Iran, has been recognized as the most equipped cancer service and referral center in the region. Thus, access to cancer diagnosis and treatment services is much better in this city than in other cities in the south of Iran. FPBCR collects data on new cancer reports from almost all diagnostic and therapeutic centers as well as death registries in Fars province. FPBCR is also the most qualified cancer registry in southern Iran in terms of completeness of case diagnosis, comparability, data quality, and timeliness [11, 12, 16]. In this study, all patients who were diagnosed with breast cancer were included as participants. Only individuals who explicitly expressed their lack of willingness to participate were excluded from the study. Based on the most recent census, the population of the FPBCR catchment area; i.e., Fars province, is more than five million people with a female: male ratio of 1:1.03. The majority of inhabitants live in urban/suburban areas. Patients’ data including age, gender, date of birth, and date of current cancer diagnosis is collected, abstracted, and computerized by well-experienced cancer registrars. In addition, topographical and morphological data of malignancies are abstracted and registered based on the third edition of the International Classification of Diseases for Oncology (ICD-O-3). Duplicated cases are identified and removed by applying software-based techniques [11, 12, 17]. An adapted version of CanReg5 software is used by FPBCR.

The present study protocol was approved by the Ethics Committee of Shiraz University of Medical Sciences (ethics code: IR.SUMS.REC.1401.115). All methods were carried out in accordance with relevant guidelines and regulations, and there was no publication of identifying information. Moreover, informed consent was obtained from all subjects and/or their legal guardian (s) at the time of registration.

### Data preparation and analysis

New cancer cases with ICD-O-3 codes C50.0-C50.9 were retrieved and prepared. New annual cases were also counted for the categories defined based on age group (under 25, 25–34, 35–44, 45–54, 55–64, 65–74, and 75 and older), morphology, and topography. Age specific incidence rates of breast cancer were calculated by dividing the number of new cases by stratum-specific mid-year population. (“Stratum-specific mid-year population” refers to the population count within specific strata or subgroups of a larger population, typically measured at the midpoint of a given year). Annual overall and truncated age-standardized incidence rates were also calculated based on the 2000 world population (Supplementary Table 2). The incidence data were truncated at 25 (considering a very small number of new cases aged younger than 25 years and assuming that younger populations are at a lower risk of breast cancer). Then, truncated incidence rates were calculated. Temporal trends of the estimated age-specific incidence rates as well as overall and truncated age-standardized incidence rates were analyzed both overall and in the most prevalent subgroup of morphology (IDC), using Joinpoint regression software (release 4.9.1). The Joinpoint regression program is a specialized software developed by the US National Cancer Institute for analyzing trends in data from the Surveillance Epidemiology and End Results Program. This powerful method identifies and connects multiple line segments on a log scale at “joinpoints” to describe changes in data trends. Significance tests are performed using a Monte Carlo permutation method. Furthermore, the program estimates the Annual Percent Change (APC) and its corresponding 95% confidence interval for each line segment. The APC is evaluated to determine if it differs significantly from the null hypothesis of no change (0%). Notably, in the final model, each joinpoint represents a statistically significant change in trends (increase or decrease), with each trend characterized by an APC value [18, 19]. As we were primarily interested in general changes of breast cancer incidence over the studied time period (2001–2018) rather than short-term changes, we chose one joint point in each regression model to avoid overwhelming readers with a large amount of statistical output. The data was prepared and analyzed using MS Office Excel and Stata software (release 14, College Station, TX: Stata Corp LLC). P value < 0.05 was considered statistically significant.

## Results

### Description

There were a total of 9, 657 breast cancer cases including 237 males (2.45%) and 9,420 females (97.55%).

### Location

Among females, after ‘Not Otherwise Specified’ (NOS), the most common location of breast cancer was the upper outer quadrant (n = 707, 7.51%). In males, most of the cases were classified as NOS and it was impossible to determine the most frequent location considering the small number of patients.

### Males

The largest number of male patients with breast cancer belonged to > 75 (n = 41, 17.2%) and 45–49 (n = 34, 14.3%) age groups (Table 1**).** In addition, the most common morphology was ICD-O code 8500, which was IDC (n = 178, 75.1%). Most of the cases with IDC belonged to 45–54 (n = 29) and > 75 (n = 29) age groups. There were no cases of breast cancer under 15 years old.

**Table 1:** Frequency of breast cancer (total and invasive ductal carcinoma) divided by age group and sex (2001–2018)

	Invasive ductal carcinoma	Total breast cancer	Invasive ductal carcinoma	Total breast cancer
Age group (years)	Males		Females	
Below 5	0	0	2	4
5–9	0	0	0	2
10–14	0	0	0	2
15–19	2	2	5	12
20–24	0	3	26	37
25–29	2	2	162	206
30–34	4	7	441	554
35–39	6	7	743	925
40–44	17	18	1,097	1,355
45–49	29	34	1,176	1,492
50–54	18	27	1,034	1,337
55–59	20	29	840	1,084
60–64	18	22	662	843
65–69	24	32	466	616
70–74	9	13	280	386
≥ 75	29	41	351	565
Total	178	237	7,285	9,420

### Females

The most frequent age group was 45–49 (n = 1492, 15.8%) in all types of female breast cancer (Table 1). Also, the age groups 40–44 years and 50–54 ranked second and third, respectively. Totally, there were7285 female patients with IDC (77.3%), most of whom belonged to the 45–49 age group (n = 1176, 16.1%). Additionally, four cases of breast cancer were under five years old, two of whom had IDC (one diagnosed in 2014 and the other in 2017).

### Trend analysis in all subtypes of breast cancer in females

**Fig. 1:**
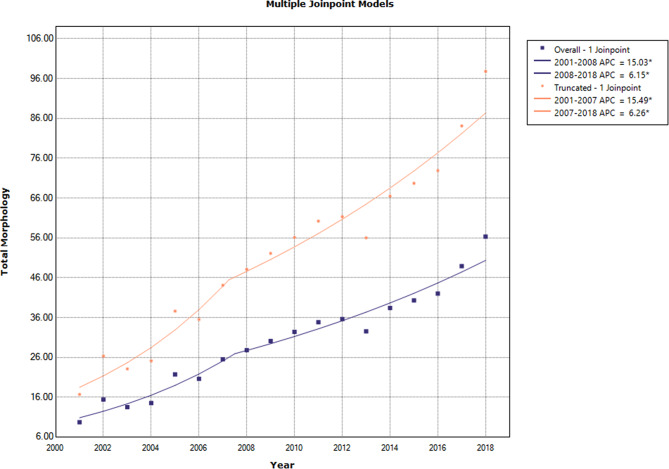
Annual percentage change of age-standardized incidence rates (overall and truncated) among females during 2001–2018

The results revealed an increasing trend in the annual overall and truncated age-standardized incidence rates of breast cancer during 2001–2018 [AAPC: 9.5 (95% CI: 7.5, 11.5) and 9.6 (95% CI: 7.6, 11.6), respectively] (Table 2). The APC of overall age-standardized incidence rates decreased from 15.03 (95% CI: 10.4, 19.8) in 2007 to 6.15(95% CI: 4.0, 8.4) in 2018. The APC of truncated age-standardized incidence rates also decreased from15.49 (95% CI: 10.8, 20.4) in 2007 to 6.26(95% CI: 4.1, 8.5) in 2018 (Fig. 1; Table 3).

**Table 2:** The results of trend analysis of age-specific rates and overall and truncated age-standardized incidence rates in all subtypes of female breast cancer during 2001–2018

Cohort	Range	Lower Endpoint	Upper Endpoint	AAPC	Lower CI	Upper CI	Test Statistic~	P-Value~
Under 25 − 0 Joinpoints	Full Range	2001	2018	26.1	-9.8	76.2	1.5	< 0.1
25–34–1 Joinpoint	Full Range	2001	2018	8.0*	4.4	11.8	4.4	< 0.1
35–44–1 Joinpoint	Full Range	2001	2018	8.8*	6.7	11.0	8.6	< 0.1
45–54–1 Joinpoint	Full Range	2001	2018	8.2*	6.0	10.4	7.6	< 0.1
55–64–1 Joinpoint	Full Range	2001	2018	11.8*	8.1	15.6	6.5	< 0.1
65–74–1 Joinpoint	Full Range	2001	2018	11.5*	8.6	14.5	8.1	< 0.1
75 + − 1 Joinpoint	Full Range	2001	2018	12.3*	7.2	17.6	4.9	< 0.1
Overall − 1 Joinpoint	Full Range	2001	2018	9.5*	7.5	11.5	9.6	< 0.1
Truncated − 1 Joinpoint	Full Range	2001	2018	9.6*	7.6	11.6	9.8	< 0.1

* Statistically significant

**Table 3:** The results of trend analysis of age-specific rates and overall and truncated age-standardized incidence rates in all subtypes of female breast cancer during 2001–2018 segmented into two distinct time periods

Cohort	Segment	Lower Endpoint	Upper Endpoint	APC	Lower CI	Upper CI	Test Statistic (t)	Prob > |t|
Under 25 − 0 Joinpoints	1	2001	2018	26.1	-9.8	76.2	1.5	0.162
25–34–1 Joinpoint	1	2001	2009	10.3*	3.5	17.5	3.3	0.005
25–34–1 Joinpoint	2	2009	2018	6.2*	1.5	11.1	2.9	0.012
35–44–1 Joinpoint	1	2001	2006	19.5*	12.0	27.5	5.9	< 0.001
35–44–1 Joinpoint	2	2006	2018	5.0*	3.4	6.6	6.8	< 0.001
45–54–1 Joinpoint	1	2001	2008	11.8*	6.9	16.8	5.4	< 0.001
45–54–1 Joinpoint	2	2008	2018	5.9*	3.5	8.3	5.5	< 0.001
55–64–1 Joinpoint	1	2001	2008	22.0*	13.4	31.4	5.8	< 0.001
55–64–1 Joinpoint	2	2008	2018	5.5*	1.7	9.6	3.1	0.008
65–74–1 Joinpoint	1	2001	2016	10.3*	8.5	12.0	13.1	< 0.001
65–74–1 Joinpoint	2	2016	2018	19.9	-1.0	45.2	2.0	0.062
75 + − 1 Joinpoint	1	2001	2006	25.0*	9.8	42.3	3.7	0.002
75 + − 1 Joinpoint	2	2006	2018	7.0*	2.3	11.9	3.2	0.006
Overall − 1 Joinpoint	1	2001	2008	15.0*	10.4	19.8	7.3	< 0.001
Overall − 1 Joinpoint	2	2008	2018	6.1*	4.0	8.4	6.2	< 0.001
Truncated − 1 Joinpoint	1	2001	2007	15.5*	10.8	20.4	7.5	< 0.001
Truncated − 1 Joinpoint	2	2007	2018	6.3*	4.1	8.5	6.3	< 0.001

* Statistically significant

**Fig. 2:**
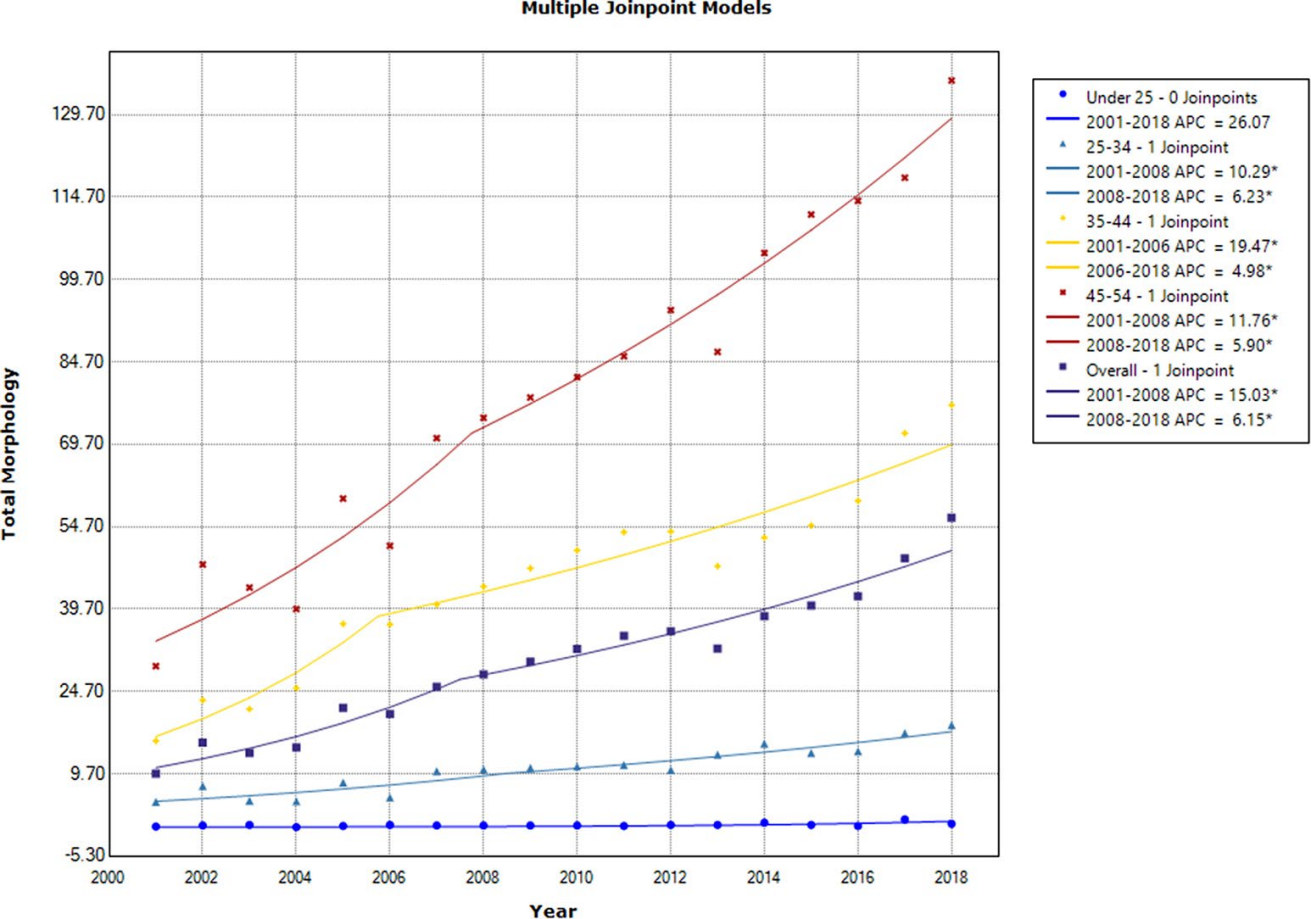
The annual percent change of age-specific and overall age-standardized incidence rates in females in the < 55 age group during 2001–2018

Breast cancer followed an ascending trend in the age groups under 55 years, except for the < 25 age group that showed a steady trend [APC of age-specific incidence rates: 26.07 (95% CI: -9.8, 76.2). Among < 55 age groups, the highest APC belonged to the 25–34 age group during 2007–2018 [APC of age-specific incidence rates: 6.23(95% CI: 1.5, 11.1)] (Fig. 2; Table 3).

**Fig. 3:**
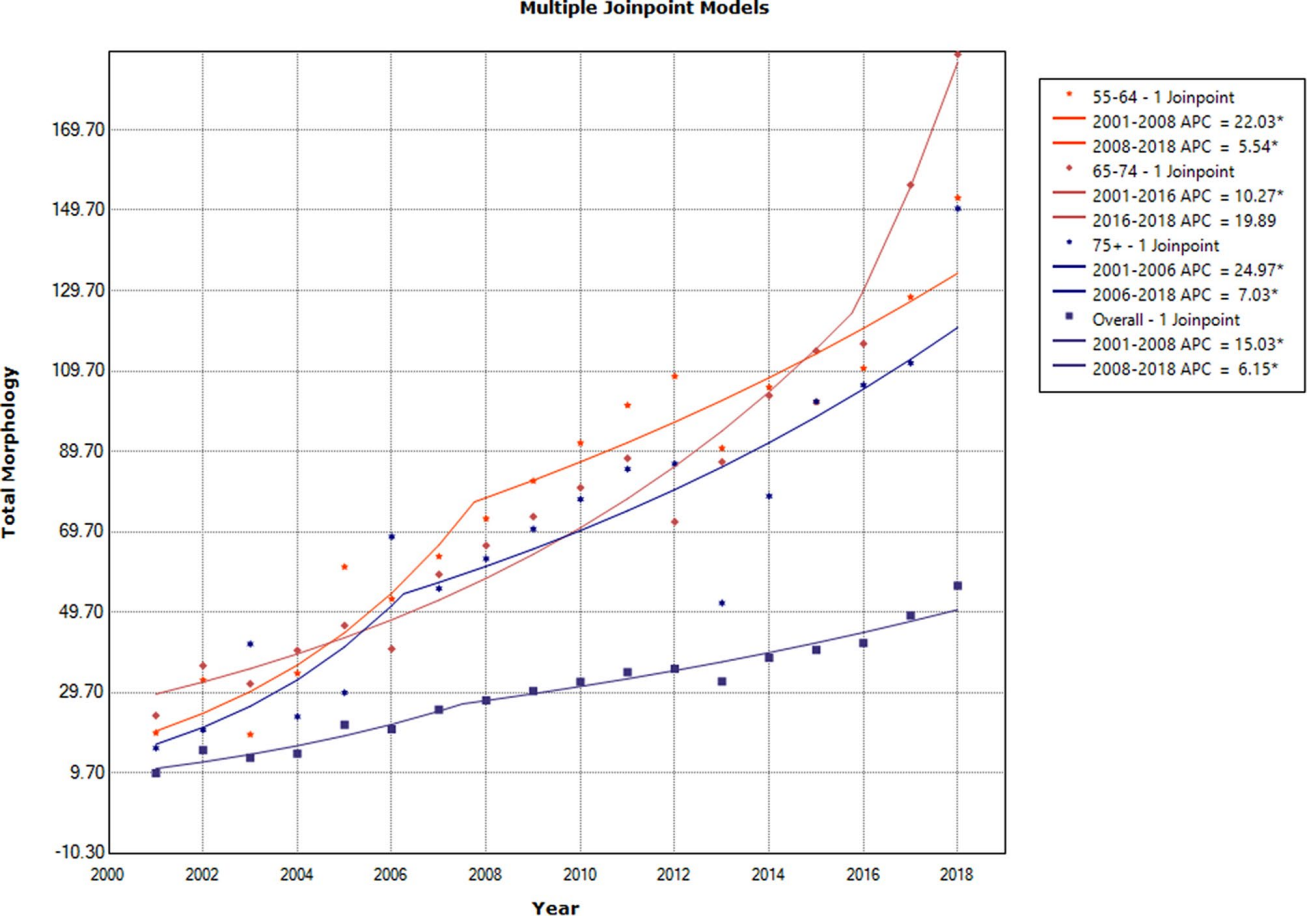
The annual percent change of age-specific and overall age-standardized incidence rates in females in ≥ 55 age groups during 2001–2018

The results revealed an increasing trend in ≥ 55 age groups, except for the 65–74 age group that showed a steady trend during 2015–2018 [APC of age-specific incidence rates: 19.89(95% CI: -1.0, 45.2)] (Fig. 3; Table 3).

### Trend analysis of invasive ductal carcinoma of the breast in females

**Fig. 4:**
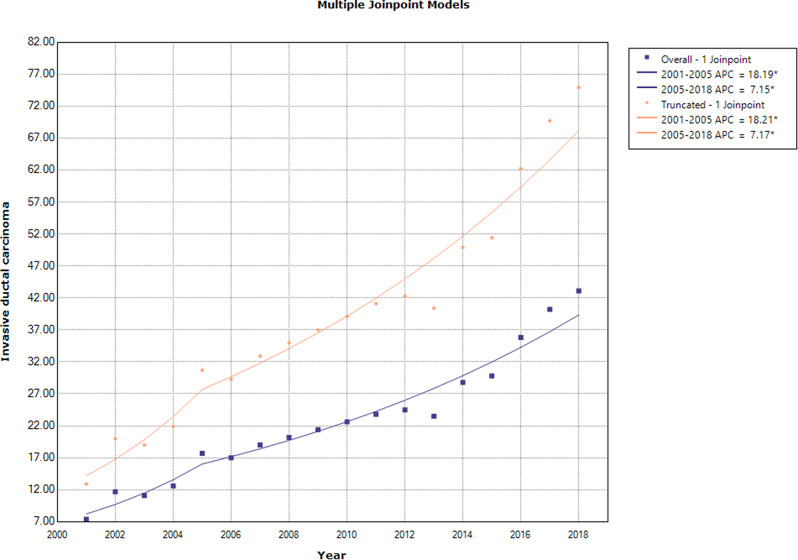
The annual percent change of age-standardized incidence rates (overall and truncated) for invasive ductal breast cancer in females during 2001–2018

The results showed an increasing trend in the annual overall and truncated age-standardized rates of IDC throughout the period of 2001–2018. [AAPC: 9.7(95% CI: 7.3, 12.0) and 9.7 (95% CI: 7.4, 12.0), respectively]. Before 2005, APC was 18.19 (95% CI: 8.2, 29.1) and 18.21 (95% CI: 8.3, 29.0) for overall and truncated age–standardized incidence rates, respectively. After 2005, these measures were respectively obtained as 7.15 (95% CI: 5.6, 8.7) and 7.17 (95% CI 5.6, 8.7) (Fig. 4**).**

**Fig. 5:**
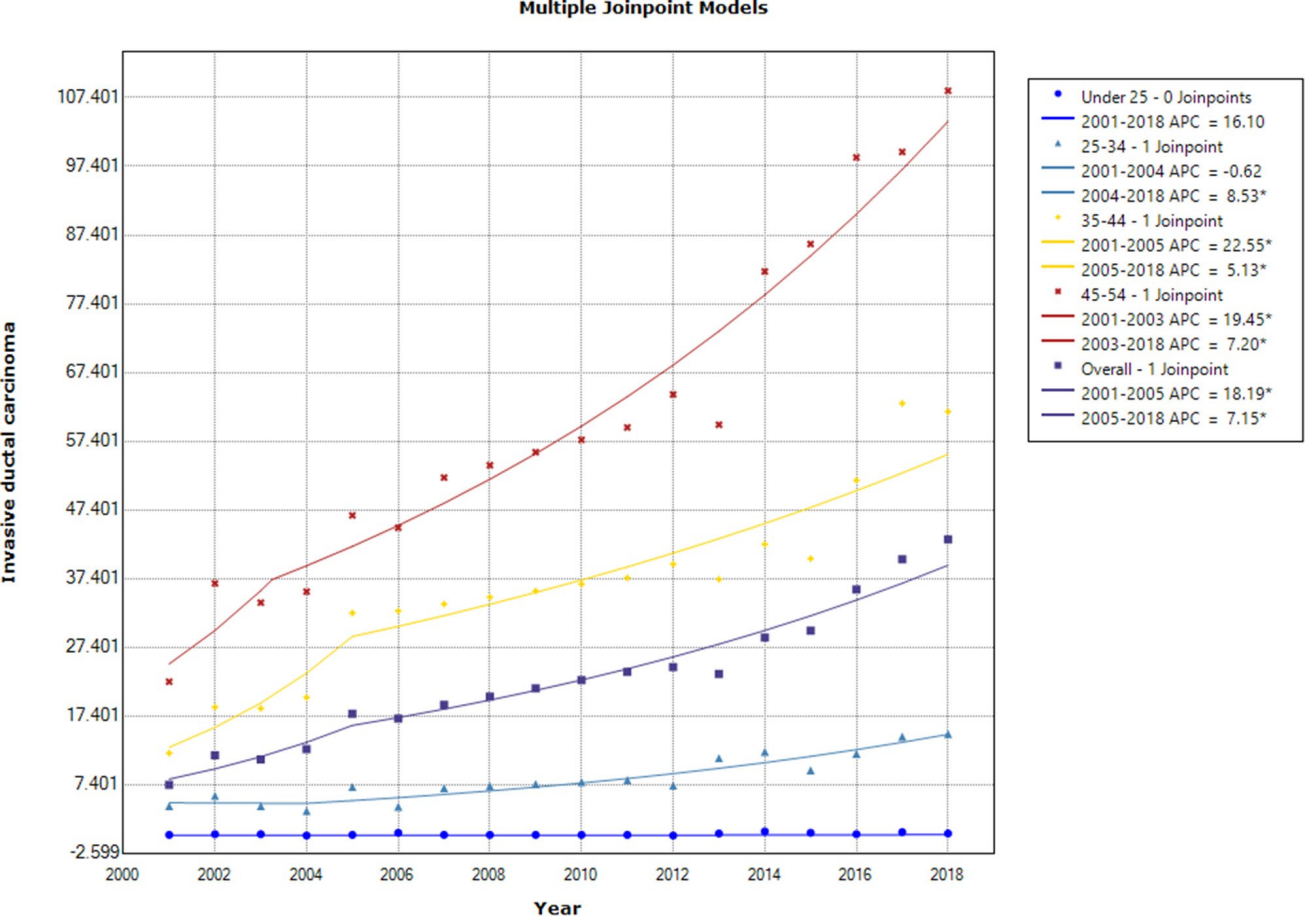
Annual percent change of the age-specific and overall age-standardized incidence rates for invasive ductal breast cancer in females in < 55 age groups during 2001–2018

The results revealed an ascending trend in the age-specific incidence rates of IDC in the age groups under 55 years, except for < 25 and 25–34 age groups that showed steady APCs during 2001–2004[16.1(95% CI: -26.8, 84.3) and − 0.6 (95% CI: -23.7, 29.4), respectively] (Fig. 5).

**Fig. 6:**
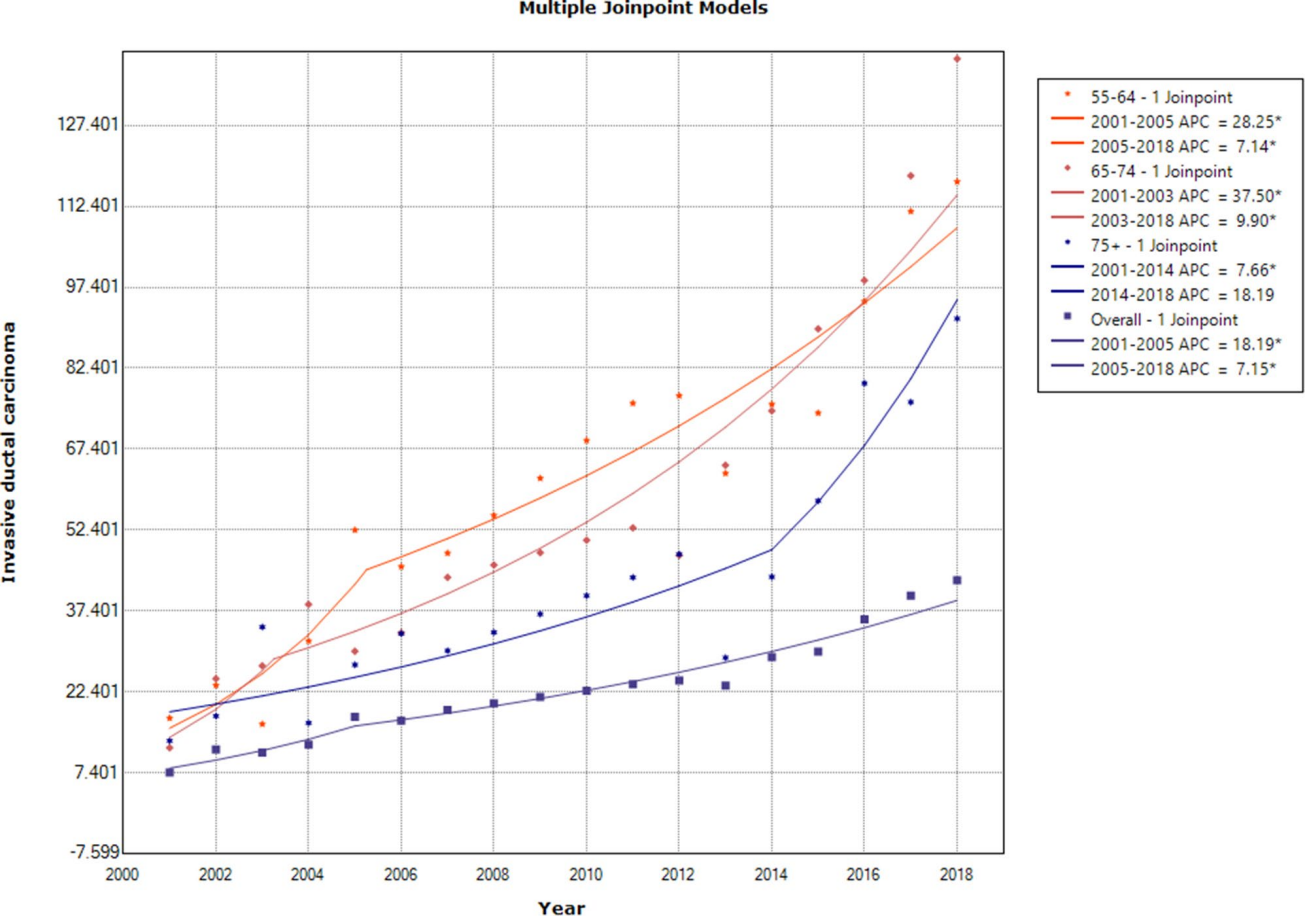
Annual percent change of the age-specific and overall age-standardized incidence rates for invasive ductal breast cancer in females in ≥ 55age groups during 2001–2018

The results indicated an ascending trend in the age-specific incidence rates of IDC in ≥ 55 age groups, except for the > 75 age group that showed a steady trend during 2014–2018 [APC: 18.19(95% CI -6.9, 50.0)] (Fig. 6).

## Discussion

The study results revealed an ascending trend in the incidence of breast cancer among females during 2001–2018, but the trend was increasing less during the recent years. The most common morphology of breast cancer was IDC and its trend was similar to the general trend of different types of breast cancer. The most common site of breast cancer was the upper outer quadrant. Most of the breast cancer cases were female and males accounted for 2.45% of breast cancer cases. In addition, the majority of female breast cancer cases belonged to the 40–55 age group.

While the incidence of breast cancer increased among women between 2001 and 2018, the rate of increase has slowed in recent years. The trend in breast cancer incidence is complex and is influenced by a variety of factors, including changes in screening practices, risk factors, and treatment modalities. One possible explanation for the recent slowdown in the rate of increase is that screening programs and women’s awareness have plateaued in recent years. It is important to continue to monitor this trend and to research the underlying causes in order to develop more effective prevention and treatment strategies. A recent study in African countries also found an increasing trend of breast cancer. However, the incidence showed different trends in different regions [20].Breast cancer also followed an ascending trend in Iraq [21], Vietnam [22], and Wales [23] during the recent years. In the US, the mortality rate of breast cancer was found to be decreasing in the > 40 age group, but not in the 20–39 age group [24].The decreased mortality rate of breast cancer in women above 40 years old in the US could result from the higher rate of screening of breast cancer cases, leading to early diagnosis and timely management of patients.

In the current research, the most common morphology was IDC whose trend was similar to the overall trend of all types of breast cancer. Besides, its trend was increasing less during the recent years compared to the past. Generally, the most prevalent form of invasive breast cancer is infiltrating ductal carcinoma, which involves the abnormal proliferation of duct cells into stroma [25]. However, the majority of cases with IDC cannot be specifically classified and are thus considered as NOS [25].

Generally, there are several risk factors for breast cancer including age at menarche, genetic susceptibility, race, and family history of breast cancer which are not modifiable. On the other hand, changeable risk factors are age at first birth, and factors associated with life style. BMI, alcohol consumption, cigarette smoking, and physical activity are the major risk factors for consideration [26]. Also, hormonal exposure, both exogenous including oral contraceptive pills and hormone replacement therapy, and endogenous hormonal conditions including age at first menarche, pregnancy and lactation periods, and age at menopause are identified risk factors for breast cancer [27]. Nulliparity, early menarche, late menopause, no lactation, and hormonal therapy were reported as risk factors for invasive ductal carcinoma [28].The diagnosis of breast cancer is usually done by breast mammogram, also sonography and MRI are the tools for diagnosis. The breast mammography can detect calcification, growth, or mass in breast tissue [29]. The sonography can majorly differentiate between breast cyst and solid masses. In case of solid mass, further evaluation is needed to confirm the diagnosis [30]. Breast MRI commonly used in high risk patients, those with strong family history or positive BRCA (Breast Cancer) gene mutation [31].

The prognosis of IDC of breast is highly related to the expression of hormone receptors mainly estrogen receptor, progesterone receptor, and HER-2 through immunohistochemistry staining. Hormone receptor positive breast cancers have better prognosis [32]. Triple negative breast cancer is one of the most aggressive type of breast cancer with no expression of estrogen receptor, progestogen receptor and HER-2 with poor prognosis [33].

The present study results indicated that the most common topographical subtype was the upper outer quadrant, which was similar to the findings of other studies [34–37]. The higher incidence of upper outer quadrant breast cancer could reflect a higher amount of breast tissue in this region [38]. Moreover, several studies have reported that the location of breast cancer could be an important prognostic factor for survival, as patients with tumors located in the upper outer quadrant of breast were found to have better survival [35]. Additionally, patients with lower inner quadrant tumors showed significantly poorer survival compared to other groups [37].

In the present study, the largest number of breast cancer cases belonged to the 40–55 age group. In some previous studies also, the 40–60 age group was reported as the most common age group for breast cancer [39, 40]. In a study performed on Iraqi females, a significant increase was observed in the incidence of breast cancer in the 50–70 age group [21]. Moreover, a global increase has been detected in the incidence of breast cancer among females in all age groups, especially the < 50 age group [41]. Notably, the incidence of breast cancer in advanced stages showed an ascending trend among females under 40 years of age in the US [24]. Furthermore, a recent meta-analysis on the Iranian population disclosed that family history, hormone replacement therapy, passive smoking, late full-term pregnancy, abortion, and sweets consumption were the risk factors of breast cancer development [42]. Fewer number of births was also associated with an increased incidence of breast cancer in females aged 40–44 years [43]. Taken together, all these risk factors might play a role in reducing the age of breast cancer occurrence amongst Iranian women.

This study is strengthened by the high generalizability of the study results due to use of data from FPBCR, which is one of the most qualified cancer registries in southern Iran. However, certain limitations should be addressed. Firstly, there was a large number of patients with unspecified topographies. Therefore, analysis of the incidence trend of location was not possible. Secondly, there was a paucity of data regarding the possible biological and clinical variables of breast cancer in the studied population like age at first birth, lifestyle factors, body mass index, parity, menarche age, menopause age, lactation history, and hormonal therapy. Hence, further analyses considering the related risk factors were not possible.

## Conclusion

The incidence of breast cancer among women living in southern Iran showed an increasing trend from 2001 to 2018. However, the rate of increase exhibited a milder slope in the more recent years. Since breast cancer was most prevalent in the 40–55 age group, our study provided important insights to reduce the age of breast cancer screening from 50 to 40 years in healthy Iranian women. However, additional studies focusing on the cost-effectiveness and potential benefits and harms of this change in policy would provide a more complete basis for implementing such a policy change. As a result, more new cases are diagnosed at earlier stages of cancer and can provide health policy makers with more accurate evidences for resource allocation and planning in preventive, diagnostic, and therapeutic dimensions.

### Electronic supplementary material

Below is the link to the electronic supplementary material.

Supplementary Material 1

Supplementary Material 2

## Data Availability

The data presented in this study are available on request from the corresponding author.
