# Supplementary material for: Investigating the trends of incidence rates of breast cancer in Southern Iran: a population based survey
Source: BMC Womens Health. 2023 Nov 10;23:589. doi: 10.1186/s12905-023-02757-7 (PMC10638837; doi:10.1186/s12905-023-02757-7)
Supplement: Supplementary file 2 — Supplementary Material 2 [file 12905_2023_2757_MOESM2_ESM.docx]

Supplementary Table 2: Age distribution of the study area and worldwide in 2000

| **Age group** | **World Population (%)** | **Fars Population (N)** |
| --- | --- | --- |
| Under 25 | 42.84 | 1,981,923 |
| 25-34 | 15.54 | 704,238 |
| 35-44 | 13.74 | 492,255 |
| 45-54 | 11.41 | 356,526 |
| 55-64 | 8.27 | 176,741 |
| 65-69 | 5.17 | 123,820 |
| 75+ | 3.06 | 80,564 |
| **Total** | 100 | 3,916,067 |
